# Two unusual cases of Gitelman’s syndrome with a complex inheritance: how the phenotype can help interpret the genotype: lesson for the clinical nephrologist

**DOI:** 10.1007/s40620-020-00861-7

**Published:** 2020-09-14

**Authors:** Lorenzo A. Calò, Viviana Palazzo, Leonardo Salviati, Franca Anglani

**Affiliations:** 1 Nephrology, Dialysis and Transplantation Unit, Kidney Histomorphology and Molecular Biology Laboratory, Department of Medicine-DIMED, University of Padova, Padova, Italy; 2 Medical Genetics Unit, Meyer Children’s University Hospital, Florence, Italy; 3 Clinical Genetics Unit, Department of Women’s and Children’s Health, University of Padova, Padova, Italy

**Keywords:** Gitelman’s syndrome, Bartter’s syndrome, Distal renal tubular acidosis, *SLC12A3*, *KCNJ1*, *SLC4A1*, Complex inheritance, Genetic modifiers, Blended phenotype

## Case 1

A 4-year-old female was referred to our hospital in 2014 for an episode of gastroenteritis and abdominal pain. Biochemical tests revealed Salmonella group B infection, and hypokalemia (2 mmol/L). Renal function was normal, as were other blood parameters. The child recovered with no complications, but hypokalemia and hypochloremia persisted (2.4 mmol/L and 95 mmol/L, respectively, at discharge) despite treatment with K supplements (10 mmol three times daily). One month later, the patient was readmitted to the Pediatric Unit for abdominal pain and cramps. Blood tests showed hypokalemia (2.0 mmol/L), hypomagnesemia (0.55 mmol/L), hypocalciuria (2.0 mmol/24 h), and renin–angiotensin–aldosterone system (RAAS) activation (renin 49.6 mIU/L, aldosterone 740 pmol/L), metabolic alkalosis, normal renal function, and no proteinuria. Blood pressure was normal to low. The patient was given K (27 mmol/L four times daily, oral suspension) and Mg (325 mg twice daily). Gitelman’s syndrome (GS) was suspected due to the patient’s biochemical (severe hypokalemia, hypomagnesemia and hypocalciuria), hormonal (RAAS activation, normo-/hypotension) and clinical (more oriented therapy based on the severe hypokalemia and mild hypomagnesemia) characteristics, despite the presence of features more often associated with Bartter’s syndrome (BS) (the severe clinical phenotype, unusual appearance at an early age and hypochloremia with mild hypomagnesemia).

**Fig. 1:**
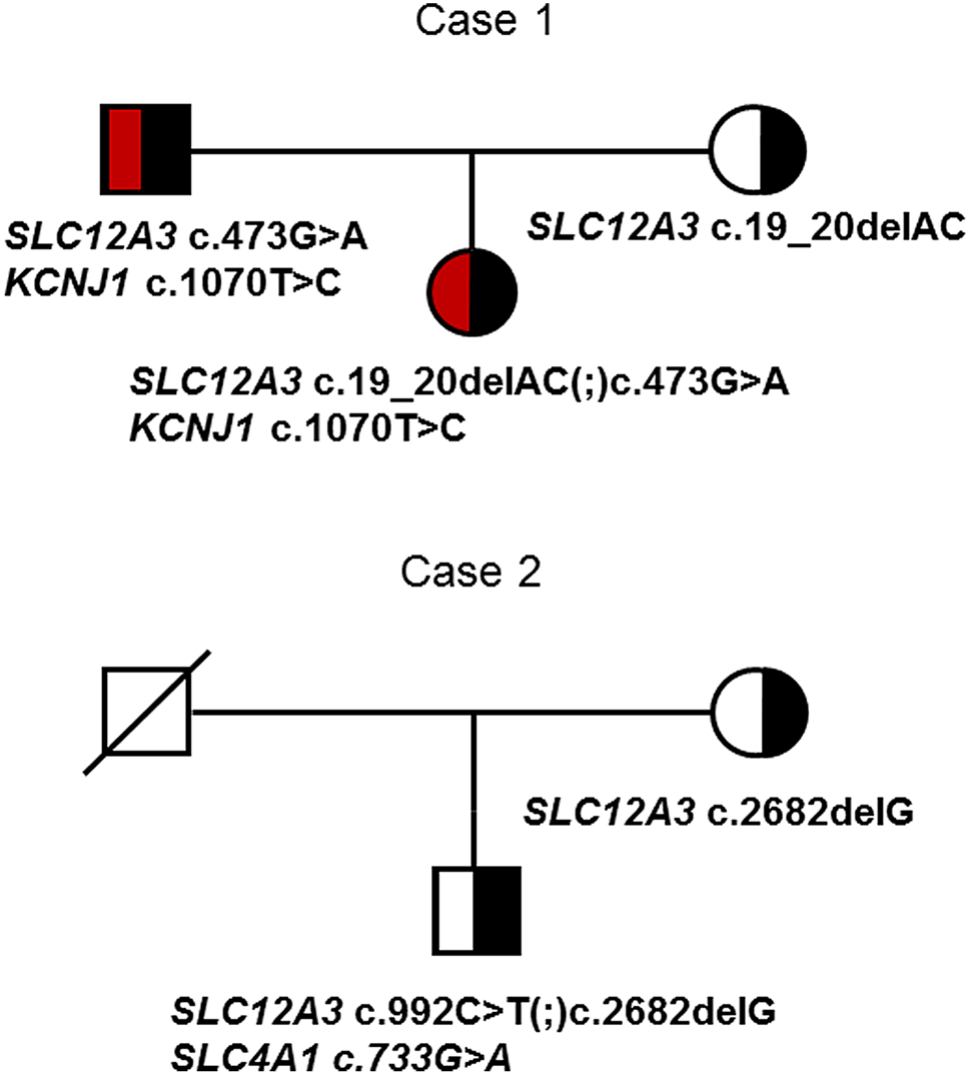
Complex inheritance in Gitelman’s syndrome. Pedigrees showing inheritance of pathogenic variants in Case 1 and Case 2

Multi‐gene panel testing was part of the routine diagnostic procedures and was used for mutational screening of BS and GS genes revealing a compound heterozygosity for two known GS-causing mutations in the *SLC12A3* gene (RefSeq NM_000339.2): the frameshift c.20_21delCA, p.(Thr7fs) variant (ClinVar ID 817609) and the c.473G>A p.(Arg158Gln) missense variant (ClinVar ID 64,769) [1], thus confirming the clinical diagnosis of GS. The diagnostic workflow detected another missense variant, c.1070T>C, p.(Met357Thr), in the *KCNJ1* gene (RefSeq NM_000220.4) encoding ROMK, the pore-forming subunit of the kidney's main potassium-secreting channel, found to be associated with severe forms of BS (BS type 2) [2, 3]. The variants were inherited from her parents (Fig. 1).

## Case 2

A 44-year-old male came to our observation with persistent hypokalemia. Biochemical analyses showed hypokalemia (2.3 mmol/L), hypomagnesemia (0.4 mmol/L), hypocalciuria (1.7 mmol/L), moderately increased creatinine (110 μmol/L), eGFR 68 ml/min, no proteinuria, normal urinalysis, modest metabolic alkalosis (HCO3^−^ 29 mmol/L), RAAS activation (renin 50 mIU/L, aldosterone 845 pmol/L), normotension (122/72 mmHg), and normal urinary electrolytes. At 10 years of age he had reportedly been diagnosed with incomplete renal tubular acidosis with potassium wasting (of which we unfortunately have no medical record). He reported no nephrolithiasis or episodes of renal colic, and no renal stones appeared on imaging. The patient was treated with K and Mg supplements (40 mmol/day and 360 mg/day, respectively). Despite the unusually modest metabolic alkalosis and moderately impaired renal function, the biochemical, hormonal and clinical findings suggested GS, so the patient was screened for GS and BS gene mutations. Clinical exome sequencing revealed two novel variants in the *SLC12A3* gene—a missense c.992C>T, p.(Pro331Leu), and a frameshift c.2682delG, p.(Lys894fs)—classified respectively as ‘likely pathogenic’ and ‘pathogenic’ according to the ACMG guidelines [Supplementary Information], prompting a molecular diagnosis of GS. Another very rare (gnomAD Minor Allele Frequency (MAF) 0.0007) missense c.733G>A, p.(Val245Met) (rs148170067) variant was also identified in the *SLC4A1* gene (RefSeq NM_000342) encoding the chloride-bicarbonate anion exchanger 1 (AE1), the pathogenic variants of which cause autosomal dominant or recessive distal tubular acidosis (dRTA) (OMIM # 179800, #611590).

## Discussion

Bartter’s syndrome (OMIM #s: type 1, 60678; type 2, 241200; type 3, 607364; type 4a, 602522; type 4b, 613090; type 5, 300971) and Gitelman’s syndrome (OMIM # 263800) are autosomal recessive tubulopathies characterized by hypokalemia, metabolic alkalosis, activation of the renin–angiotensin–aldosterone system, high levels of angiotensin II (Ang II), but normo- or hypotension, and blunted Ang II cardiovascular effects. GS also involves hypomagnesemia and hypocalciuria [2, 3]. These syndromes are caused by biallelic pathogenic variants in genes encoding proteins involved in renal electrolyte homeostasis: the *KCNJ1, SLC12A1, CLCNKB, BSND, CLCNKA* + *CLCNKB*, *MAGED2* genes for BS; and the *SLC12A3* gene for GS [2]. The three main clinical variants are: classic BS; neonatal BS; and GS. Defects in genes affecting transport channels in Henle’s ascending loop cause classic and neonatal BS, while in GS the defect occurs in transport channels of the distal convoluted tubule. GS is less severe than BS.

The two patients herein described presented unusual GS phenotypes that were difficult to interpret clinically and to treat because variants were detected not only in the *SLC12A3* gene, but also in other genes involved in BS and distal tubulopathies.

### What is the clinical significance of the missense variant in the KCNJ1 gene in case 1?

The Human Genome Mutation database (HGMD ID CM960893) reports the *KCNJ1* variant as a cause of BS because it was first described as a heterozygous mutation in a BS patient [4]. The same variant was later found in compound heterozygosity in a child with benign BS type 2 [5]. The frequency of this variant was recently estimated to be < 1% (rare variant), but around 1% in the non-Finnish European population (gnomAD MAF 0.01155) (rs59172778), and 19 homozygotes have been identified. Furthermore, in vitro studies by Schwalbe et al. [6] have also raised doubts about its functional significance. These two latter pieces of information indeed suggest excluding this variant as pathogenic, as previously proposed.

### Could the KCNJ1 gene be a modifier gene?

With the introduction of next-generation sequencing in the diagnostic workflow, we have been discovering more patients with complex inheritance, thus allowing us a greater understanding of how complex interactions between allelic and locus heterogeneity may affect disease phenotypes, particularly when dealing with genes working in associated pathways, as are BS and GS genes. More and more examples of hereditary disorders with oligogenic inheritance (pathogenic variants in more than one gene) have been described [Supplementary Reference 1]. From these studies, it appears evident that no genetic variant acts alone, i.e. some other variants (genetic modifiers) may lessen or worsen the disease, resulting in the variability of phenotypic outcomes. The unusual severity of GS phenotype encountered in our patient led us to hypothesize that the missense variant can act as a genetic modifier by exacerbating the severity of the disease and by inducing BS-like clinical manifestations.

### What is the clinical significance of the missense variant in the SLC4A1 gene in case 2?

The dRTA clinical phenotype varies considerably among patients, but typically includes chronic metabolic acidosis, hypokalemia, abnormally alkaline urine, nephrocalcinosis, and nephrolithiasis [7]. The clinical signs of dominant dRTA (ddRTA) are generally milder than those of the autosomal recessive type, and the disease presents later on. The *SLC4A1* missense variant we identified was judged deleterious using in silico tools but had never been associated with dRTA before. As for the inheritance of the three variants, the *SLC12A3* frameshift was inherited from the mother. The father could not undergo genetic testing because he had died of myocardial infarction at 58 years of age, and no clinical data were available regarding any renal disease. We therefore cannot say whether the *SLC4A1* variant was inherited from the father or is de novo. It is, instead, likely that the second *SLC12A3* variant was inherited from the father (Fig. 1).

Some questions could be raised about the pathogenic significance of the rare *SLC4A1* variant. It is not located at the COOH-terminus of the protein, or in the integral membrane domains where most of the mutations causing ddRTA have been detected so far—although a mutation in the AE1 N-terminal H1 domain (p.Arg388Cys) was recently described in ddRTA [8, 9]. Since the highly-conserved portion of the *SLC4A1* gene across species begins at the Phe379 residue [9], the variant we detected (being located at the Val245 residue) seems less likely to cause ddRTA. Indeed, according to the ACMG guidelines the variant could be classified as a variant of uncertain significance. The patient’s phenotype could help interpret these genotype data.

### Is the patient’s phenotype a blend of autosomal recessive GS and autosomal ddRTA?

Finding variants in two different genes responsible for two different monogenic disorders raises questions on how to interpret the phenotype. Unlike digenic inheritance, in which pathogenic variations at two specific loci contribute to the manifestation of a single disease [Supplementary Reference 2], dual (or multiple) molecular diagnoses combine separate diagnoses of more than one independently-segregating genetic locus [Supplementary Reference 3], and the phenotypic complexity of the latter can present a challenge to the physician.

The blending of two distinct disease phenotypes in a single patient may suggest an apparently new clinical entity. Alternatively, molecular diagnoses with two overlapping disease phenotypes may be interpreted as the phenotypic expansion of a single disease.

Our patient’s medical history (he was clinically diagnosed with incomplete hypokalemic dRTA at 10 years of age) supports the hypothesis of a dual molecular diagnosis and hence of a blended phenotype.

Since dRTA shares some features with GS, the patient’s phenotype might also be interpreted as an expansion of the GS phenotype. The patient’s juvenile expression of dRTA and his less marked metabolic alkalosis associated with an unusual CKD in adulthood may support this second hypothesis suggesting that the *SLC4A1* variant is probably hypomorphic. By acting as a genetic modifier rather than a disease-causing variant, it lent the patient’s phenotype some unusual clinical features.

### Did genetic diagnosis change the clinical management of these patients?

The complex inheritance of the two cases we have described as provided by the genetic analysis, although not requiring a substantial change of the usual pharmacological approach to GS which is based on K and Mg supplements, did however lead to some changes in the clinical management, in particular for the Case 1 patient. As mentioned above, she presented with a more severe phenotype, which required higher doses of K and MG supplementation than usual and stricter clinical follow-up with more frequent visits. No further measures were instead adopted for the Case 2 patient. Both patients are currently in good condition and continue their follow-up every 6 months.

## Electronic supplementary material

Below is the link to the electronic supplementary material.

Supplementary file1 (DOCX 18 kb)

## Data Availability

The data supporting the findings of this study are available from the corresponding author.
