# Supplementary material for: Two unusual cases of Gitelman’s syndrome with a complex inheritance: how the phenotype can help interpret the genotype: lesson for the clinical nephrologist
Source: J Nephrol. 2020 Sep 14;34(4):1327–30. doi: 10.1007/s40620-020-00861-7 (PMC8357653; doi:10.1007/s40620-020-00861-7)
Supplement: Supplementary file 1 — Supplementary file1 (DOCX 18 kb) [file 40620_2020_861_MOESM1_ESM.docx]

**Two unusual cases of Gitelman syndrome with complex inheritance: how phenotype can help interpretation of the genotype: lesson for the clinical nephrologist**

^1^Lorenzo A Calò, ^2^Viviana Palazzo, ^3^Leonardo Salviati, ^1^Franca Anglani

**Journal of Nephrology**

**Corresponding author**

Dr. Franca Anglani

Nephrology, Dialysis and Transplantation Unit

Kidney Histomorphology and Molecular Biology Laboratory

Department of Medicine – DIMED

University of Padua

Via Giustiniani n° 2

35128 Padova (Italy)

phone: +390498212155

email: franca.anglani@unipd.it

ORCID: orcid.org/0000-0003-1534-4458

**Supplementary information**

*DNA extraction*

Written informed consent was obtained from the patient . Peripheral blood DNA was extracted using QIAamp Mini Kit (QIAGEN®, Hilden, Germany), according to manufacturer’s instructions, and quantified by NanoDROP 2000 Spectrophotometer (Thermo Scientific, Waltham, MA, USA).

*DNA library preparation and Sequencing*

To construct DNA libraries we used a strategy based on enzymatic fragmentation to produce dsDNA fragments followed by End repair, A-tailing, adapter ligation and library amplification (Kapa Biosystems, Wilmington, MA). Libraries were hybridized with the protocol SeqCap EZ Exome v3 (Nimblegen, Roche, Basel, Switzerland) and sequenced by NextSeq 500 (Illumina Inc., San Diego, CA).

*Assembly, Variant Calling*

The reads were aligned with the human reference hg19 genome using Burrows-Wheeler Aligner (BWA) (1), mapped and analyzed with the IGV software (Integrative Genome Viewer, 2013 Broad Institute) (2). Downstream alignment processing (i.e. alignment sorting, indexing, deduplication and base quality score recalibration) was performed with the Genome Analysis Toolkit Unified Genotyper Module (GATK) (3) SAMtools (4) and Picard Tools (http://picard.sourceforge.net/). The GATK was used to obtain a set of raw single nucleotide variant (SNV) calls, which were filtered using the following criteria: QD < 5.0, DP < 5, FS > 60.0, QUAL < 30.0. Variants were annotated using Annovar tool (5) to obtain information such as variant frequency in different populations and the predictions of the variant effect using different methods (SIFT, Polyphen2, MutationTaster, MutationAssessor, FATHMM and FATHMM MKL).

Variants were classified and scored in agreement with the interpretation guidelines of the American College of Medical genetics and Genomics (ACMG) (6).

In details, we selected only non-synonymous, short insertion/deletion or splice-site variants (30 bp splice acceptor, 30 bp splice donor) with the following characteristics:

- variants not present or with a minor allele frequency ≤ 0.01 for autosomal recessive (AR) and with a minor allele frequency ≤ 0.001 for autosomal dominant (AD)-transmitted genes in population database “1000 Genomes Project”, “Exome Variant Server” (dbSNP147), ExAC, gnomAD.

-Variants predicted as damaging by at least 4 over 6 in silico tools (Polyphen-2, SIFT, Mutation Taster, FATHMM, FATHMM MKL, Mutation Assessor).

- variants were researched in a database that reported disease-causing mutations, we used ClinVar and the Human Gene Mutation Database (HGMD) Professional (http://www.hgmd.cf.ac.uk/ac/index.php) updated to 2019.

- variants correctly segregating within the family or representing de novo variants.

- different cut-off values for the ratio of novel allele to reference allele reads were used. For analyses of homozygous variants, we included variants with at least 80 % novel allele reads. Heterozygous variants were included only when called in at least 10 % and at most 80 % of all reads. .

Considering missense variants, we only included variants with a CADD-value of at least 10 (other variants are considered benign). The remaining variants were inspected with the Integrative Genomics Viewer to exclude sequencing errors in repetitive regions. - variants of all individuals were analyzed for known genes compiled from OMIM, regarding the inheritance model and the phenotype of each individual.

*References*

1. Li H, Durbin R (2009) Fast and accurate short read alignment with Burrows-Wheeler transform. Bioinformatics 25: 1754-1760. <https://doi.org/10.1093/bioinformatics/btp324>

2. Thorvaldsdóttir H, Robinson JT, Mesirov JP (2013) Integrative Genomics Viewer (IGV): high-performance genomics data visualization and exploration IGV. Brief Bioinform 14:178-192. <https://doi.org/10.1093/bib/bbs017>

3. McKenna A, Hanna M, Banks E, Sivachenko A, Cibulskis K, Kernytsky A, Garimella K, Altshuler D, Gabriel S, Daly M, De Pristo MA (2010) The Genome Analysis Toolkit: a MapReduce framework for analyzing next-generation DNA sequencing data. Genome Res 20: 1297-1303. <http://www.genome.org/cgi/doi/10.1101/gr.107524.110>

4. Li H, Handsaker B, Wysoker A, Fennell T, Ruan J, Homer N, Marth G, Abecasis G, Durbin R (2009) 1000 Genome Project Data Processing Subgroup: The sequence alignment/map (SAM) format and SAMtools. Bioinformatics 25: 2078-2079. <https://doi.org/10.1093/bioinformatics/btp352>

5. Wang K, Li M, Hakonarson H (2010) ANNOVAR: functional annotation of genetic variants from high-throughput sequencing data. Nucleic Acids Res 38: e164. <https://doi.org/10.1093/nar/gkq603>

6. Richards S, Aziz N, Bale S, Bick D, Das S, Gastier-Foster J, Grody WW, Hegde M, Lyon E, Spector E, Voelkerding K, Rehm HL (2015) Standards and guidelines for the interpretation of sequence variants: a joint consensus recommendation of the American College of Medical Genetics and Genomics and the Association for Molecular Pathology. Genet Med 17:405-24. <https://doi.org/10.1038/gim.2015.30>

**Supplementary references**

1. Riordan JD, Nadeau JH (2017) From Peas to Disease: Modifier Genes, Network Resilience, and the Genetics of Health. Am J Hum Genet 101:177-19. <https://doi:10.1016/j.ajhg.2017.06.004>

2. Schäffer AA (2013) Digenic inheritance in medical genetics. J Med Genet 50:641-52. <https://doi.org/10.1136/jmedgenet-2013-101713>

3. Posey JE, Harel T, Liu P, Rosenfeld JA, James RA, Coban Akdemir ZH, Walkiewicz M, Bi W, Xiao R, Ding Y, Xia F, Beaudet AL, Muzny DM, Gibbs RA, Boerwinkle E, Eng CM, Sutton VR, Shaw CA, Plon SE, Yang Y, Lupski JR (2017) Resolution of Disease Phenotypes Resulting from Multilocus Genomic Variation. N Engl J Med 376:21-31. <https://doi.org/10.1056/NEJMoa1516767>
